# Improved Intrusion Detection Based on Hybrid Deep Learning Models and Federated Learning

**DOI:** 10.3390/s24124002

**Published:** 2024-06-20

**Authors:** Jia Huang, Zhen Chen, Sheng-Zheng Liu, Hao Zhang, Hai-Xia Long

**Affiliations:** 1 College of Information Science Technology, Hainan Normal University, Haikou 571158, China; hj221204@hainnu.edu.cn (J.H.); cz221201@hainnu.edu.cn (Z.C.); lsz231209@hainnu.edu.cn (S.-Z.L.); 2 Key Laboratory of Data Science and Smart Education, Ministry of Education, Hainan Normal University, Haikou 571158, China; 3 College of Tourism, Hainan Normal University, Haikou 571158, China

**Keywords:** industrial internet of things, network intrusion detection, federated learning, data privacy

## Abstract

The security of the Industrial Internet of Things (IIoT) is of vital importance, and the Network Intrusion Detection System (NIDS) plays an indispensable role in this. Although there is an increasing number of studies on the use of deep learning technology to achieve network intrusion detection, the limited local data of the device may lead to poor model performance because deep learning requires large-scale datasets for training. Some solutions propose to centralize the local datasets of devices for deep learning training, but this may involve user privacy issues. To address these challenges, this study proposes a novel federated learning (FL)-based approach aimed at improving the accuracy of network intrusion detection while ensuring data privacy protection. This research combines convolutional neural networks with attention mechanisms to develop a new deep learning intrusion detection model specifically designed for the IIoT. Additionally, variational autoencoders are incorporated to enhance data privacy protection. Furthermore, an FL framework enables multiple IIoT clients to jointly train a shared intrusion detection model without sharing their raw data. This strategy significantly improves the model’s detection capability while effectively addressing data privacy and security issues. To validate the effectiveness of the proposed method, a series of experiments were conducted on a real-world Internet of Things (IoT) network intrusion dataset. The experimental results demonstrate that our model and FL approach significantly improve key performance metrics such as detection accuracy, precision, and false-positive rate (FPR) compared to traditional local training methods and existing models.

## 1. Introduction

The Industrial Internet of Things (IIoT), as a crucial component of modern industrial systems, is rapidly transforming operations in multiple fields, such as manufacturing, energy management, and supply chain monitoring [1,2,3]. By embedding intelligent sensors and controllers in industrial equipment and systems, the IIoT enables real-time data collection, processing, and analysis, thereby optimizing operational efficiency, reducing costs, and increasing productivity. However, with the rapid development and widespread application of the IIoT, its security threats are also increasing, especially with frequent network intrusion events, severely threatening the security operation of industrial systems and data integrity [4,5].

Network intrusion detection systems (NIDSs) play a crucial role in preventing malicious attacks and ensuring network security [6]. In recent years, there has been a growing interest in utilizing deep learning techniques for implementing network intrusion detection. However, the requirement for large-scale datasets in deep learning often becomes a limiting factor in its practical application [7]. For some devices, their local datasets may be limited, directly affecting the training effectiveness of models. To address this challenge, some approaches propose aggregating local datasets from devices for deep learning training. However, this practice may raise privacy concerns because sensitive information from devices could potentially be exposed to unauthorized personnel during data transmission [8]. Therefore, in researching network intrusion detection systems, it is essential to balance data security and model effectiveness to ensure that systems protect IIoT security while respecting user privacy rights.

Therefore, proposing a model that can protect user privacy while leveraging resources from various clients to construct a model with excellent intrusion detection performance is crucial. This holds significance for both industrial practitioners and policymakers in the IIoT security domain [9]. For industrial practitioners, enhancing the detection accuracy of potential intrusion behaviors in industrial networks will assist them in better protecting their systems and devices from unauthorized access and malicious attacks. Reducing false-positive rates will minimize disruptions and unnecessary work burdens for industrial practitioners. Improving privacy protection capabilities will contribute to enhancing system security and reliability, thereby reducing potential production interruptions and losses. For policymakers, an effective research outcome represents technological innovation in the field of IIoT security. Policymakers can promote the development of security technologies in the industrial sector by supporting and encouraging the research and application of such technologies, thereby enhancing the national level of information security. Overall, research in this area has a positive impact on both industrial practitioners and policymakers in the IIoT security domain, providing more accurate and efficient intrusion detection capabilities and facilitating technological innovation, data privacy protection, as well as cross-sector collaboration and information sharing.

Given the importance of addressing the aforementioned challenges and conducting research in this area, this paper proposes an innovative approach that combines federated learning and a novel deep learning intrusion detection model to improve the accuracy of network intrusion detection and privacy protection in IIoT environments. Specifically, the main contributions of this paper include

(1) Development of a novel deep learning intrusion detection model: A deep learning model based on deep variational autoencoders and convolutional neural networks with attention mechanisms (DVACNNs) is designed specifically for network intrusion detection in IIoT environments. The model can effectively process and analyze complex data from IIoT systems, improve the accuracy of identifying various network attacks, and incorporate deep variational autoencoders to enhance data privacy protection further.
(2) Construction and implementation of a federated learning framework: A FL framework is developed to allow multiple IIoT nodes to jointly train and optimize an intrusion detection model while preserving data privacy. This distributed learning approach not only enhances the generalization capability of the model but also provides a new approach to addressing data silos and strengthening data privacy protection.
(3) Adoption of data augmentation techniques to mitigate the problem of low precision caused by data imbalance.

Below is the organization of the subsequent sections in this paper. Section 2 reviews the relevant literature, including IIoT network security, intrusion detection methods based on deep learning, and intrusion detection methods based on both deep learning and federated learning. Section 3 provides detailed explanations of the proposed federated learning framework and the design and implementation of the deep learning intrusion detection model in this study. Section 4 demonstrates the experimental design, dataset description, and evaluation metrics. Section 5 discusses the experimental results and model performance. Finally, Section 6 summarizes the main findings of this study and proposes future research directions.

## 2. Related Work

The Industrial Internet of Things combines traditional industrial systems with cutting-edge information technology, driving the development of smart manufacturing and industrial automation. However, as the number of IIoT devices increases and their application scope expands, the network security challenges they face are also growing. Attackers may utilize various means to launch malicious attacks, such as denial of service (DoS), remote-control attacks, and data leaks [10,11,12], posing serious threats to the stable operation of industrial systems and data security. Therefore, strengthening IIoT network security, especially developing efficient network intrusion detection technology, has become a hot topic in research [13,14].

### 2.1. Deep Learning-Based Intrusion Detection Methods

Network intrusion detection technology is crucial for maintaining network security, primarily by monitoring and analyzing network traffic to identify potential malicious activities and attacks. With the advancement of machine learning and deep learning technologies, numerous studies have focused on using these techniques to enhance the performance of network intrusion detection systems, especially in terms of accuracy and efficiency when dealing with large-scale and complex data.

For example, in 2020, Ismail et al. [15] conducted research on electricity theft attacks in smart grid cyber–physical systems (CPSs) and proposed a deep learning-based detection system utilizing deep feedforward neural networks, recurrent neural networks (RNNs), and convolutional recurrent neural networks. In the same year, Keshk et al. [16] explored the application of long short-term memory (LSTM) models in anomaly detection. At the same time, in 2021, Ferrag et al. [17] studied a deep learning-based distributed denial of service (DDoS) attack detection system, employing convolutional neural networks (CNNs), deep neural networks (DNNs), and RNN models and testing them on the CIC-DDoS2019 and TON_IoT datasets. In 2022, Kumar et al. [18] proposed an attention mechanism-based deep-gated recurrent neural network (A-DGRNN) for threat intelligence-driven intrusion detection.

In summary, there has been significant research on intrusion detection methods based on deep learning, which have demonstrated high detection accuracy, thus proving the effectiveness of deep learning in the field of network intrusion detection. However, most studies still employ centralized approaches to handle large-scale datasets, which may not only raise privacy concerns but also complicate the process of improving model structures or creating new datasets, making it difficult to adapt to real-world network environments. The application of federated learning not only effectively addresses the limitations of datasets but also, to a certain extent, ensures the privacy and security of network traffic data.

### 2.2. Deep Learning and Federated Learning-Based Intrusion Detection Methods

Federated learning is an innovative distributed machine learning method that allows multiple participants to jointly train a machine learning model without sharing raw data. This approach is particularly suitable for addressing cross-domain data privacy and security issues, making it increasingly popular in network security, especially network intrusion detection. Through federated learning technology, various IIoT nodes can collaborate to train intrusion detection models with stronger performance and better generalization capabilities while protecting sensitive data. This not only improves the accuracy of model detection but also effectively addresses the problem of data silos, opening up new directions for network security research.

For example, in 2021, Tang et al. [19] developed an FL-based network intrusion detection method that addressed privacy protection issues when handling intrusion detection datasets. They employed gated recurrent unit (GRU) deep learning models for local execution on participating nodes. At the same time, a central server was responsible for aggregating and updating the model parameters of each node. This iterative training method ensures data privacy and the utilization of the collective intelligence of federated learning. Their experiments, conducted on the CICIDS2017 intrusion detection dataset, demonstrated the effectiveness and practicality of the approach.

In 2022, Tabassum et al. [20] proposed FEDGAN-IDS, a federated learning intrusion detection system combining generative adversarial networks (GANs) and federated learning. The system deploys GAN models distributed on various IoT devices, and locally generated data are enhanced through GANs, improving the effectiveness of model training and classifier accuracy. The experimental results showed that FEDGAN-IDS outperformed traditional standalone intrusion detection systems in both convergence speed and accuracy, demonstrating the potential of federated learning in complex network environments.

Also, the same year, Driss et al. [21] described an FL framework for network attack detection in vehicle sensor networks (VSNs). The framework utilizes a combination of random forests (RFs) and gated recurrent units, improving attack detection efficiency and enabling multiple devices to share computing resources and data through federated learning, effectively defending against network attacks without exposing sensitive information. This demonstrates that federated learning is not only applicable to traditional network environments but also demonstrates strong adaptability and advantages in specific industry applications.

While federated learning provides a certain degree of privacy protection by allowing clients to keep their data on their own devices without uploading them to a central server, it faces the risk of privacy leakage due to increasing attack methods such as gradient leakage attacks [22], where attackers can reconstruct original data by obtaining model parameters.

To address these issues, this paper proposes the DVACNN-Fed model framework. Even in scenarios where there is a limited amount of client data, this study demonstrates the capability to train high-performance models while ensuring data privacy.

## 3. Proposed Method

This section systematically introduces the working mechanism of the proposed DVACNN and federated learning (DVACNN-Fed) and elaborates on the designed DVACNN intrusion detection model.

### 3.1. Workflow of the DVACNN-Fed Framework

**Figure 1:**
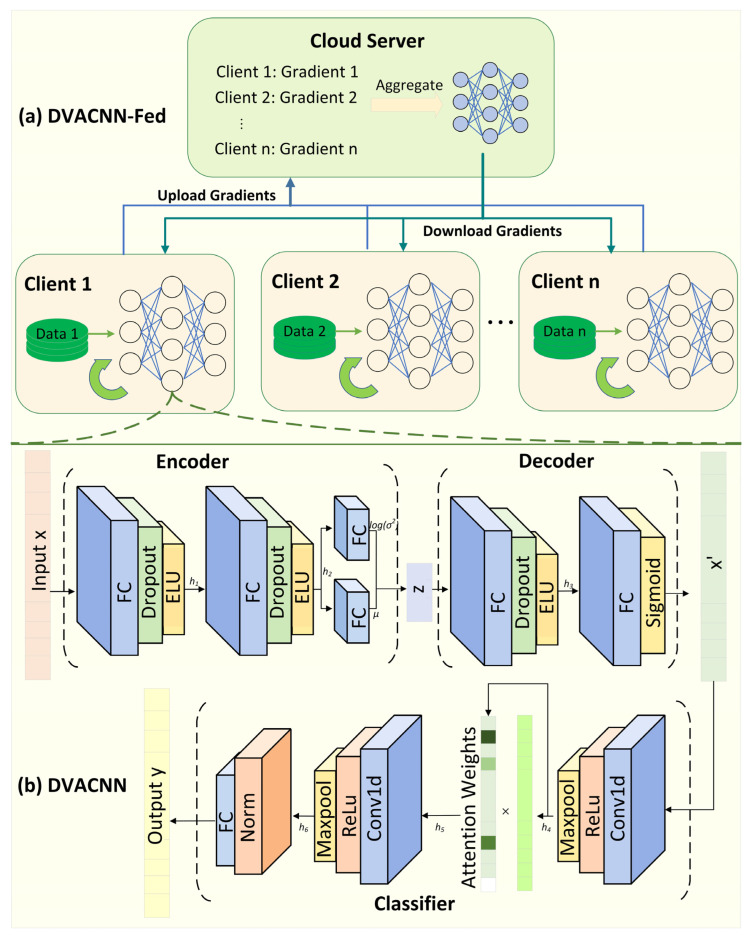
DVACNN-Fed architecture.

The DVACNN-Fed Framework aims to integrate multiple clients from various industrial domains to construct the DVACNN intrusion detection model collectively through a federated network. Figure 1a illustrates the overall architecture of federated learning in the Industrial Internet of Things environment. The operation workflow of this Framework is divided into the following six key stages:

(1) Cloud server initializes parameters: The cloud server sets a set of initial DVACNN model parameters *w*_0_, and other parameters related to model training, such as the learning rate *η*, loss function *L*, and batch size *B*.
(2) Industrial local client model training: Each client obtains the initial model parameters *w*_0_ and *η*, *L*, and *B* from the cloud server and trains the DVACNN model locally using its private data resources *D_i_* (where *i* ∈ *N* = {1, 2, …, *n*}).
(3) Industrial local client model parameters upload: After completing local training, each client processes the updated model parameters using differential privacy protection measures and then uploads them to the cloud server.
(4) Cloud server model parameter aggregation: The server collects the parameters uploaded by each client and aggregates these parameters to update the global model.
(5) Industrial local client model parameter update: Clients receive the updated global model parameters from the cloud and apply them to their local models.
(6) Iterative optimization: Clients conduct new rounds of local training based on the global model parameters. This iterative process continues until the model performance reaches the expected standard or meets the stopping conditions, such as reaching a set threshold for the number of iterations or no longer achieving significant performance gains. Consequently, the final global model is formed and can be deployed in practical application scenarios for tasks such as prediction and classification.

### 3.2. The DVACNN-Based Intrusion Detection Model

This section elaborates on the new intrusion detection model based on the deep variational autoencoder and convolutional neural network (DVACNN), as shown in Figure 1b. The model integrates a CNN module with a deep variational autoencoder (DVAE) and an ensemble attention mechanism to enhance the efficiency and accuracy of identifying network anomalies.

The DVAE is an unsupervised learning model based on directed probabilistic graphs. Traditional variational autoencoders (VAEs) [23] encode input x into new data using a set of weighted parameters. In contrast to traditional VAEs, DVAE approximates the posterior probability of neural networks, utilizing weights extracted from latent features of the data to generate new data. Due to the fact that the data inputted into the classifier are encoded data from the DVAE, and the model parameters of the encoder and decoder are not uploaded to the server, even if the parameters of the federated learning model are subjected to gradient leakage attacks during transmission, what the attacker reconstructs are the generated data after encoding through the DVAE, rather than the original data. This enhances the privacy protection performance of client-side original data. This technique significantly enhances the data privacy performance of the model without sacrificing detection accuracy and maximally defends against inference attacks. Compared to traditional encoder models, the DVAE captures underlying data features and generates new data. The DVAE model mainly consists of an encoder composed of fully connected (FC) layers, dropout layers, and exponential linear units (ELUs) activation functions, as well as a decoder-comprising FC layers, dropout layers, ELU activation functions, and sigmoid functions. 

The CNN module includes two convolutional blocks, attention weights, normalization processing (Norm), and fully connected layers. Each convolutional block consists of convolutional layers, activation functions, and max-pooling layers. 

In this model, numerical features of network traffic data are represented by a one-dimensional feature vector x and are first processed through fully connected layers. This process can be mathematically described as $FC\left(x\right)=w_{i}x+b_{i}$, where $w_{i}$ represents the weight matrix and $b_{i}$ represents the corresponding bias term. Subsequently, the model introduces the dropout mechanism, which suppresses overfitting by randomly setting the outputs of some neurons to zero. The processed data are then passed through the ELU activation function to obtain the intermediate layer output $h_{1}$; the detailed definition of this function is shown in Equation (3). Then, $h_{1}$ undergoes another series of fully connected layers, Dropout, and ELU processing to obtain the output $h_{2}$; the detailed calculation process is shown in Equations (1) and (2).

(1) $$h_{1}=ELU\left(Dropout\left(FC_{1}\left(x\right)\right)\right)$$

(2) $$h_{2}=ELU\left(Dropout\left(FC_{2}\left(h_{1}\right)\right)\right)$$

(3) $$ELU\left(x\right)=\{\begin{array}{c} x\;\;\;\;\;\;\;\;\;\;\;\;\;\;\;\;\;\;\;\;\;\;\;\;\;if\;x>0\\ \alpha \left(e^{x}-1\right)\;\;\;\;\;\;\;\;\;if\;x\leq 0 \end{array}$$

Taking $h_{2}$ as input to the fully connected layer yields the mean vector *uu* and the logarithm of the variance vector $\log\left(\sigma^{2}\right)$. The latter is transformed into the standard deviation vector σ through an exponential function. Subsequently, sampling based on u and σ produces the latent vector z. The detailed process is described in Equation (4). The random vector ε is sampled from the standard normal distribution N(0,I).

(4) $$\begin{array}{c} u=FC_{3}\left(h_{2}\right),\;and\;\log\left(\sigma^{2}\right)=FC_{4}\left(h_{2}\right)\\ \sigma =\exp\left(\frac{1}{2}\log\left(\sigma^{2}\right)\right)\\ z=u+\varepsilon \times \sigma \end{array}$$

After obtaining the latent vector z, it is sequentially input into the fully connected layer of the decoder, followed by Dropout and ELU modules to obtain intermediate output $h_{3}$. Through the sigmoid function, the output data $x^{\prime }$ are compressed within the (0, 1) interval, resulting in reconstructed data $x^{\prime }$ with the same dimensions as the input x. The detailed process is described in Equation (5).

(5) $$\begin{array}{c} \begin{array}{c} h_{3} \end{array}=ELU\left(Dropout\left(FC_{5}\left(z\right)\right)\right)\\ x^{\prime }=FC_{5}\left(Sigmoid\left(h_{3}\right)\right) \end{array}$$

In existing research, convolutional neural networks [24] were initially utilized primarily in fields such as image processing, where the input data are typically in the form of two-dimensional matrices, such as images. As a deep feedforward neural network type, the CNN consists of multiple nonlinear feature transformation layers, with parameters of these layers optimized through methods like gradient descent. In the model proposed in this study, the one-dimensional feature vector $x^{\prime }$ generated by the DVAE, is first input into a one-dimensional convolutional layer, which extracts local features of the input data through multiple filters. Following convolution, the output data undergo the rectified linear unit (ReLU) activation function to introduce nonlinearity, enabling the model to capture more complex feature representations. Subsequently, the output activated by ReLU undergoes max-pooling to obtain output $h_{4}$. This process reduces feature dimensions and preserves the most important features, effectively reducing the number of model parameters, preventing overfitting, and enhancing the model’s generalization ability. The relevant process is elaborated in Equation (6).

(6) $$h_{4}=MaxPool\left(ReLU\left(Conv1D_{1}\left(x^{\prime }\right)\right)\right)$$

To further enhance the model’s focus on critical information, this study introduces an attention weight mechanism. The role of this mechanism is to enable the model to automatically learn and adjust its focus on input data. In IIoT data, intrusions often present themselves with anomalous or abrupt characteristics. The attention mechanism aids the model in identifying and capturing key intrusion-related features. Specifically, it can concentrate attention on anomalous or suspicious data points, thereby significantly improving model performance. The mathematical representation of this step is shown in Equation (7), where α represents attention weights calculated through $\alpha =softmax\left(FC\left(h_{4}\right)\right)$. The softmax function is used to normalize attention scores, determining the weight of each feature.

(7) $$h_{5}=\alpha \times h_{4}$$

Subsequently, the feature $h_{5}$ weighted by the attention mechanism is sent to the second one-dimensional convolutional layer for finer feature extraction. After passing through the ReLU activation function again, the data are sent to the max-pooling layer. This step further abstracts features and reduces dimensionality, helping to strengthen the feature extraction of the model and generalization capabilities, as shown in Equation (8).

(8) $$h_{6}=MaxPool\left(ReLU\left(Conv1D_{2}\left(h_{5}\right)\right)\right)$$

**Figure 2:**
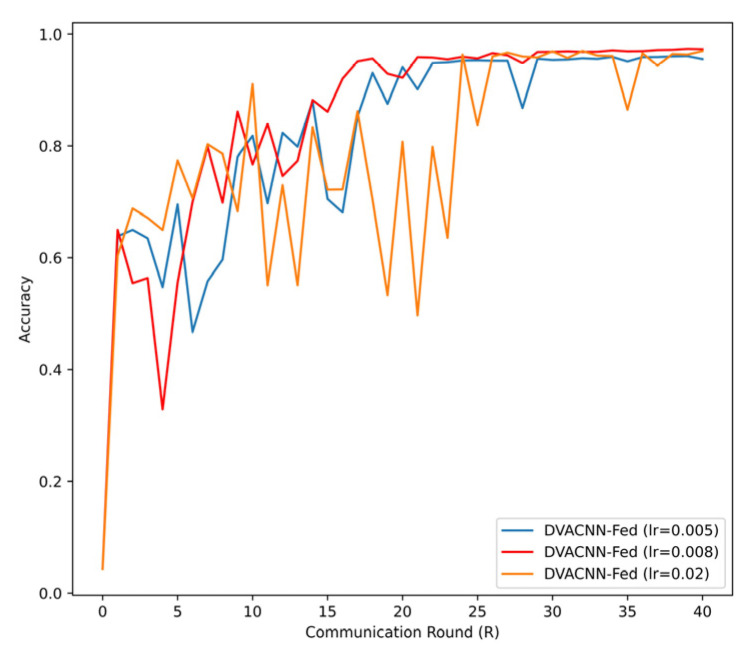
Test accuracy under a different learning rate (lr) on TON-IoT dataset.

Next, the data flow to the normalization layer for standardization, which is crucial for accelerating model convergence and improving stability. After normalization, the data enter the fully connected layer, which is weighted and integrated for the final classification decision. The output y of the fully connected layer is the final output of the model used to complete the classification task. The detailed calculation process is shown in Equation (9). This series of complex operations is comprehensively demonstrated in Figure 2.

(9) $$y=FC_{5}\left(Norm\left(h_{6}\right)\right)$$

The model employs a cross-entropy loss function to measure the difference between the predicted output of the model and the actual class and to optimize this difference into a scalar loss value. The specific formula is as follows:

(10) $$L=-\frac{1}{N}\sum_{i=1}^{N}\sum_{j=1}^{C}y_{ij}\log\left({\hat{y}}_{ij}\right)$$

where N is the sample size, C is the number of classes, $y_{ij}$ is the true label (0 or 1) of the *i*-th sample belonging to the *j*-th class, and ${\hat{y}}_{ij}$ is the predicted probability of the *i*-th sample belonging to the *j*-th class.

## 4. Experiment

### 4.1. Datasets

A series of tests were conducted in this study to validate the effectiveness of the proposed intrusion detection module. Two relatively recent IoT-based public datasets, TON_IoT [25] and BoT-IoT [26], were selected.

The TON_IoT dataset represents an Industry 4.0 and Industrial IoT collection. Data collection involved gathering data from various sources such as mobile devices, smart TVs, and host systems. These data were used to establish connections between IoT and IIoT devices, supervise processes and physical gateways (e.g., routers and gateways), and assess the vulnerability of public PHP websites to potential attacks. This dataset comprises 43 features, covering the latest attacks in the Industrial IoT.

The BoT-IoT dataset was generated in the Network Experimentation Lab at the University of New South Wales, Canberra, to simulate real network environments. Simulated network traffic was generated using the Ostinato tool and Node-Red (for non-IoT and IoT traffic). In this network environment, normal traffic and botnet traffic were combined. Data files were grouped based on attack types and subcategories to simplify labeling. This method utilized 5% of the original dataset, containing independent training and testing files with 18 features.

Preprocessing methods such as feature mapping, feature selection, and feature normalization were applied for both datasets. Regarding feature selection, 19 and 10 features were chosen using the Pearson correlation coefficient (PCC) technique described in [27].

Considering the issue of imbalanced data samples [28], imbalanced data in machine learning can lead to model bias toward the majority class, resulting in poor performance on the minority class. It also affects performance evaluation and feature learning, potentially causing overfitting and sampling bias issues. To address this problem, SMOTE-ENN was employed for data augmentation to improve the classification accuracy of minority classes. This method combines the ability of SMOTE synthetic instance generation [29] (for minority classes) and ENN [30], which excludes specific observations from both classes that are markedly different from the observed class and its nearest majority class.

### 4.2. Experimental Environment

The experiments were conducted on a computer equipped with an Intel Core i9 processor (3.0 GHz), 128 GB RAM, and an NVIDIA GeForce RTX 4090 GPU. The experimental operating system was Windows 10. The model was implemented using Python and developed and trained using the PyTorch framework (version 3.8.0). 

### 4.3. Experimental Setup

In the experiment, the selection of hyperparameters often significantly influences the performance of a model. For instance, the hyperparameters in regularization terms can significantly affect the model’s generalization ability. Proper regularization can effectively prevent overfitting and improve the model’s performance on unseen data. After multiple experiments, this study ultimately performed 20 global communication rounds and 20 local iteration cycles, with a batch size 50. All experiments utilized the stochastic gradient descent (SGD) optimizer [31] with a learning rate of $8\times 10^{-3}$. In this paper, we focused on analyzing the impact of the learning rate on model training. According to the observation results from Figure 2, when the learning rate is set to 0.005, the model’s convergence speed is relatively slow; when the learning rate is 0.2, the model’s performance fluctuates greatly, with a slower convergence speed. However, when the learning rate is set to 0.008, the model demonstrates the best convergence speed and stability. It is evident that setting the learning rate too small or too large significantly affects the model’s performance. Therefore, appropriate hyperparameter selection is crucial for the model’s performance.

For the TON_IoT dataset, 80% was used for training and 20% for testing. Additionally, the training data were evenly partitioned among 20 industrial clients for local model training. The BoT-IoT training and testing sets were stored as two independent files. Due to the large scale of the training dataset, approximately one-third of the training data were selected for this experiment and divided among 20 clients to simulate scenarios with less local data. All trained deep learning models were evaluated on the same test data.

### 4.4. Evaluation Metrics

This experiment employed two metrics to evaluate privacy protection performance. The first is the privacy index ($P_{index}$) [27], which evaluates the level of privacy protection by comparing the difference between the reconstructed and original data. It is calculated as follows:

(11) $$P_{index}=\frac{\left(Var\left(R\right)-Var\left(D\right)\right)}{Var\left(R\right)}$$

Here, R represents the original data, and D represents the transformed data. The higher the value of $P_{index}$, the higher the level of privacy protection. 

The second metric is information loss (IL) [27], which estimates the rate of information loss that occurs during the reconstruction process due to computing the density function $R_{x}$. Here, $R_{x}$ represents the density function of the original data feature x, and $D_{x}$ represents the density function of the reconstructed data feature x. It is calculated as follows:

(12) $$IL=\frac{1}{4}E\left[\int_{\Omega x}|R_{x}-D_{x}|dx\right]$$

Here, E is the expected value operator, and Ωx is the intersection of the domains of $R_{x}$ and $D_{x}$. A higher value of IL indicates less retained original feature information, thereby implying a higher level of privacy protection.

Regarding the evaluation of model classification performance, accuracy, precision, recall, F1-score, false-positive rate (FPR), ROC curve, and PR curve were used. These metrics depend on four terms: true positives (TPs), true negatives (TNs), false negatives (FNs), and false positives (FPs). These terms correspond to the sample quantities where positive samples are correctly predicted as positive, negative samples are correctly predicted as negative, positive samples are incorrectly predicted as negative, and negative samples are incorrectly predicted as positive, respectively. 

Accuracy: The proportion of samples correctly classified by the model for a given test dataset.

(13) $$Accuracy=\frac{TP+TN}{TN+FN+TP+FP}$$

Precision: The proportion of true positive samples among the samples predicted as positive.

(14) $$Precision=\frac{TP}{TP+FP}$$

Recall: The proportion of positive samples predicted as positive out of all positive samples, also known as true-positive rate (TPR).

(15) $$Recall=\frac{TP}{TP+FN}$$

F1-score: A combination of precision and recall. Precision and recall are mutually exclusive; when one increases, the other decreases. To reconcile these two metrics, the F1-score is introduced:

(16) $$F1-score=2\times \frac{Precision\times Recall}{Precision+Recall}$$

False-positive rate (FPR): The proportion of samples incorrectly predicted as positive out of all true negatives, also referred to as the false acceptance rate (FAR). Lower values are preferable.

(17) $$FPR=\frac{FP}{TN+FP}$$

Receiver operating characteristic (ROC) curve: The acceptability curve. Points on the curve reflect responses to the same signal stimulus but are obtained under different judgment criteria. The curve connects points with FPR on the X-axis and TPR on the Y-axis. Generally, the closer the ROC curve is to the top-left corner, the better the model performance.

Precision–recall (PR) curve: Shows the precision variation at different recall rates. Generally, the closer the PR curve is to the top-right corner, the better the model performance.

## 5. Experimental Results

**Figure 3:**
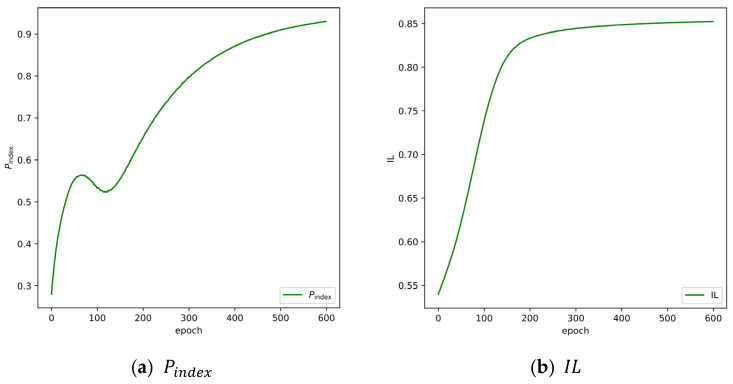
The performance of privacy protection on BoT-IoT datasets.

To validate the privacy protection performance of the model, Figure 3 presents the evaluation results of two privacy metrics on the BoT-IoT dataset. As depicted, both metrics achieved favorable results, with a $P_{index}$ of 94.26% and an IL of 85.24%. 

### 5.1. Comparative Experiment Introduction 

The comparative experiments in this paper are mainly divided into two aspects: the comparison of classification models and the comparison with models built solely locally and under ideal conditions.

In the comparison of classification models, the DVACNN-Fed is compared with state-of-the-art methods. For instance, Keshk et al. [16] introduced the application of the LSTM model in anomaly detection. LSTM is a special recurrent neural network capable of learning sequential dependencies in sequence prediction tasks, often exhibiting good performance. Additionally, Chen et al. [32] used a CNN-based federated framework for data classification, consisting of two convolutional layers, two max-pooling layers, two fully connected layers, and a softmax layer. Moreover, Ilango et al. [33] proposed a feedforward convolutional neural network. Furthermore, Kumar et al. [18] proposed the A-DGRNN model, which utilizes attention-based deep-gated recurrent neural networks (A-DGRNNs) for threat intelligence-driven detection to detect unauthorized intrusions.

In addition to comparing with the above models, the performance of intrusion detection models built solely locally with limited data resources was experimentally evaluated and compared with the performance of an ideal model constructed by a central entity utilizing data resources from all clients. 

### 5.2. Performance Comparison with State-of-the-Art Studies

**Figure 4:**
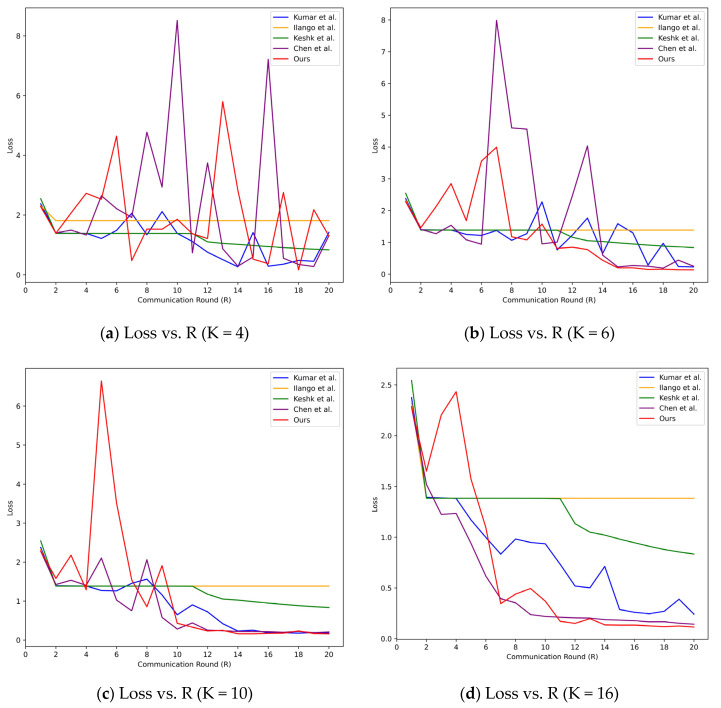
Test loss curves of considered intrusion detection models under four scenarios on TON-IoT datasets.

The performance of the proposed DVACNN approach in this paper is compared with the baseline studies [14,15,16]. Four sets of experiments were conducted, considering client numbers (K) of 4, 6, 10, and 16, and the experiments were conducted on two datasets. The following are the experimental results on the TON_IoT dataset:

From the loss curves in Figure 4, it can be observed that regardless of the value of K, the loss of the DVACNN model stabilizes when the training epochs reach 20. Thus, the proposed model exhibits rapid convergence. Meanwhile, the loss curve of the DVACNN exhibits a smoother descent, indicating its higher efficiency and robustness.

**Figure 5:**
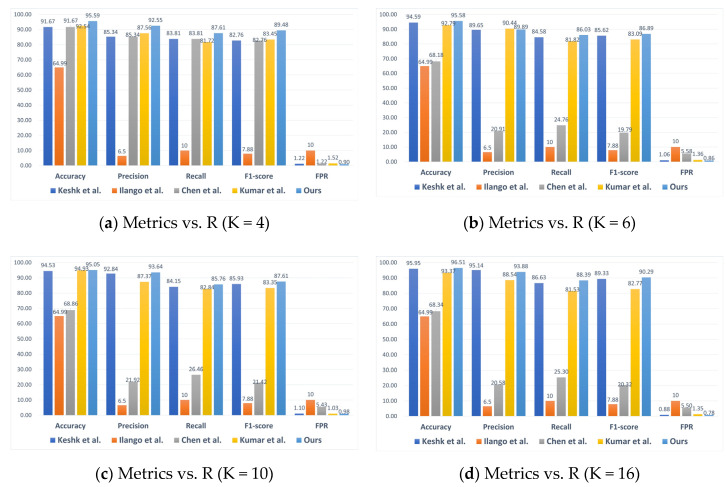
Comparison of considered intrusion detection models under four scenarios on TON-IoT datasets.

At this point, all evaluation metrics reached their optimal levels. Figure 5 shows the numerical performance results of the model in terms of accuracy, precision, recall, F1-score, and FPR under four scenarios of client numbers (K = 4, 6, 10, and 16). It can be observed that the DVACNN model outperforms all other state-of-the-art models on all metrics. When K = 4, the accuracy, precision, recall, F1-score, and FPR of the DVACNN are 95.59%, 92.55%, 87.61%, 89.48%, and 0.009, respectively. Compared to most control experiments, the accuracy, precision, recall, and F1-score increased by approximately 3%, 7%, 4%, and 6%, respectively, while the FPR decreased by 0.003. When K = 6, 10, and 16, the model proposed in this paper still performs the best, with accuracy, precision, recall, F1-score, and FPR metrics as follows: 95.58%, 89.89%, 86.03%, 86.89%, and 0.0086; 95.05%, 93.64%, 85.76%, 87.61%, and 0.0098; 96.51%, 93.88%, 88.39%, 90.29%, and 0.0078. It can be seen that the performance of this model is good regardless of the number of clients.

**Figure 6:**
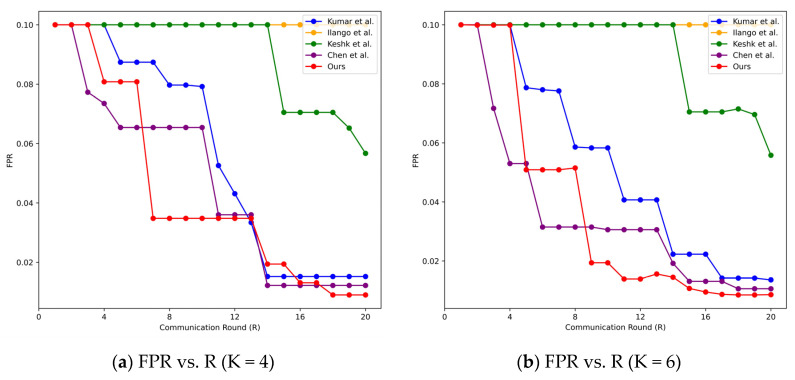

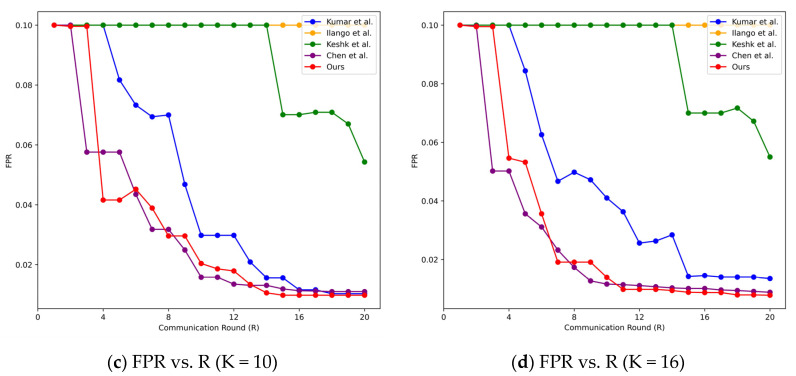
FPR of considered intrusion detection models under four scenarios on TON-IoT datasets.

From Figure 6, it can be observed that as the communication round (R) increases from 1 to 20, the performance of each intrusion detection model gradually improves. When R is sufficiently large, the FPR reaches its optimal value and gradually stabilizes. 

**Figure 7:**
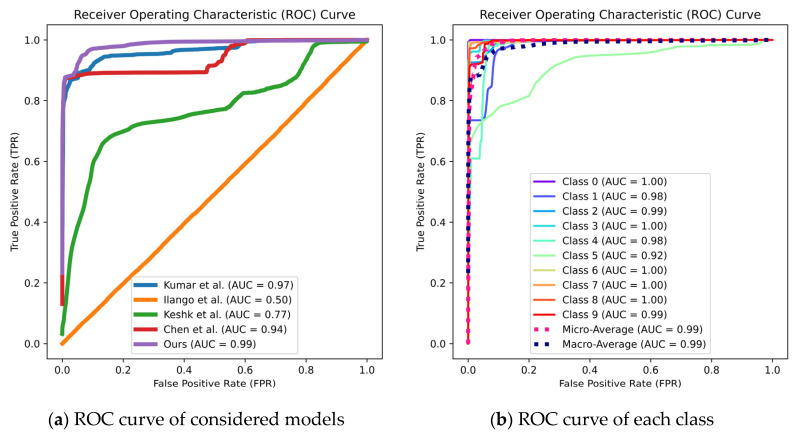

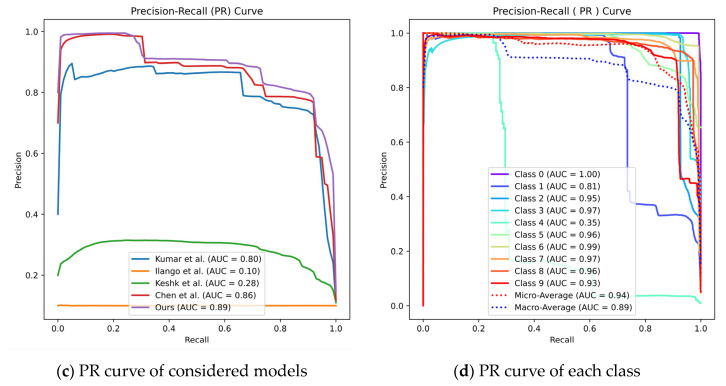
ROC and PR curves of considered intrusion detection and each class on TON-IoT datasets.

Figure 7a,c show the ROC curves and PR curves of different models corresponding to K = 6, while Figure 7b,d show the ROC curves and PR curves of different classes and the macro- and micro-averages of the DVACNN model. It can be seen from the figures that the AUC values of the ROC and PR curves of the DVACNN model are the highest, with the ROC curve AUC values for most classes above 0.98 and the PR curve AUC values for most classes above 90%, indicating excellent classification performance of the model. 

**Figure 8:**
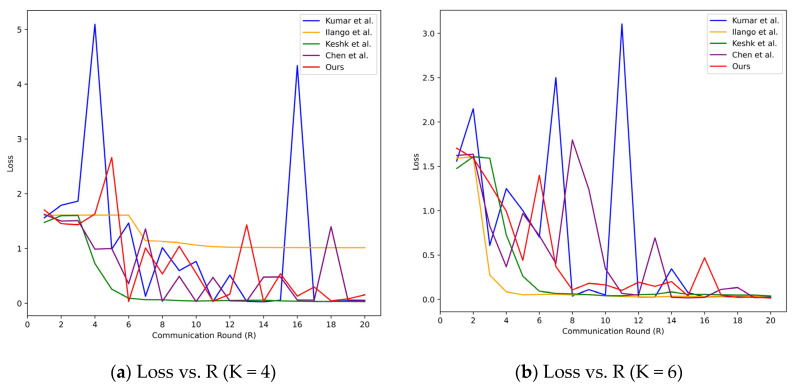

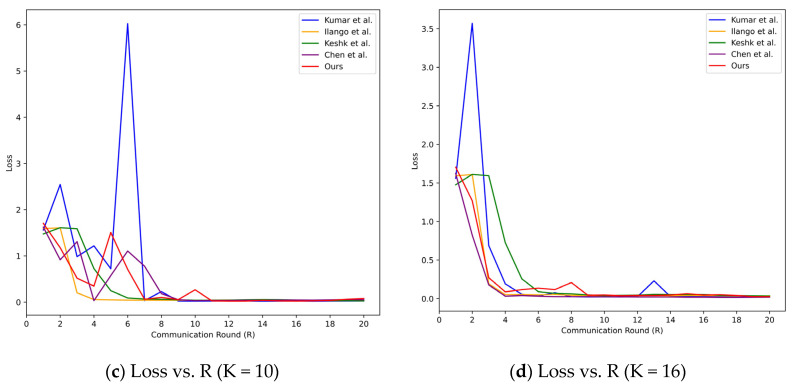
Test loss curves of considered intrusion detection models under four scenarios on BoT-IoT datasets.

**Figure 9:**
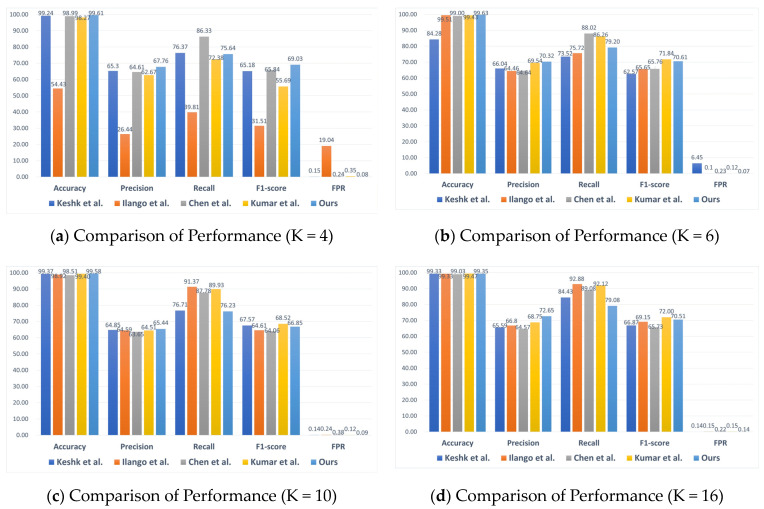
Comparison of considered intrusion detection models under four scenarios on BoT-IoT datasets.

From the BoT-IoT dataset, it can be observed in Figure 8 that when reaching the 20th round, the model losses in different scenarios are almost close to zero and tend to stabilize. At this point, the evaluation metrics of each model are shown in Figure 9. The proposed model achieves the best accuracy, precision, and FPR levels in all four scenarios, indicating the model’s good generalization ability. Specifically, all accuracies are above 99.35%. Although the precision is slightly lower due to the imbalance of samples, it remains above 65% and is higher than that of the comparative models. The results for FPR are also excellent, all below 0.0014, indicating better performance of this model compared to the FPR values of the control experiments. In summary, whether on the TON-IoT dataset or the BoT-IoT dataset, this model performs exceptionally well, indicating its strong generalization ability and robustness. Considering the representativeness of these two datasets in the field of the Internet of Things, as well as the excellent results achieved by the model on them, it can be inferred that the model possesses good scalability.

### 5.3. Performance Comparison with Local and Ideal Models 

**Figure 10:**
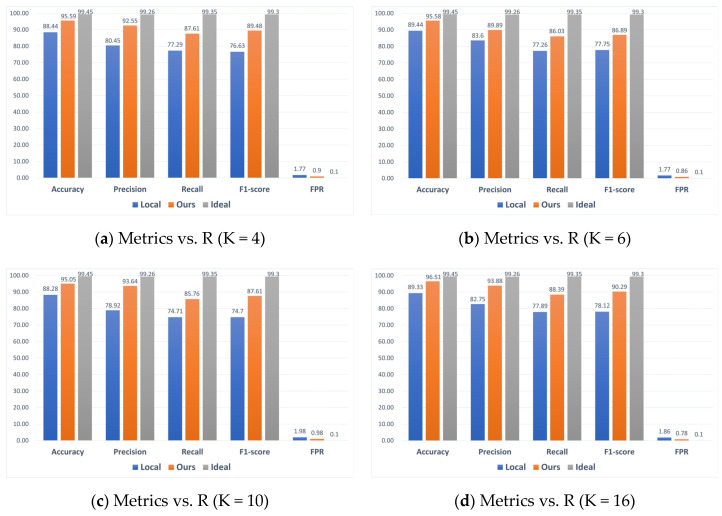
Comparison of local, ideal, and ours under four scenarios on TON-IoT datasets.

In addition to the above experiments, the performance of intrusion detection models built locally for each client with limited data resources and an ideal model constructed by a central server utilizing all data resources was evaluated. Figure 10 shows the numerical results of the single-client, ideal model and federated learning-based model on all five metrics for different settings of K values on the TON-IoT dataset. It can be seen that compared to single-client detection models, the results based on federated learning are better. Although the performance of the federated learning scheme is slightly lower compared to the ideal model, it still performs well, considering the difficulty of achieving the ideal environment.

**Figure 11:**
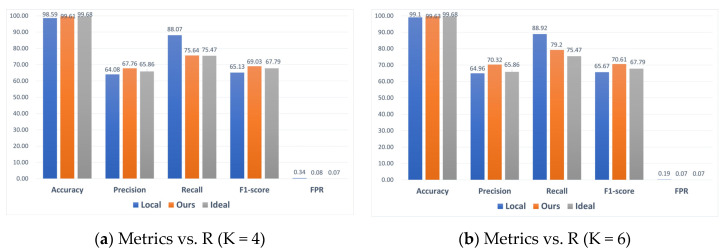

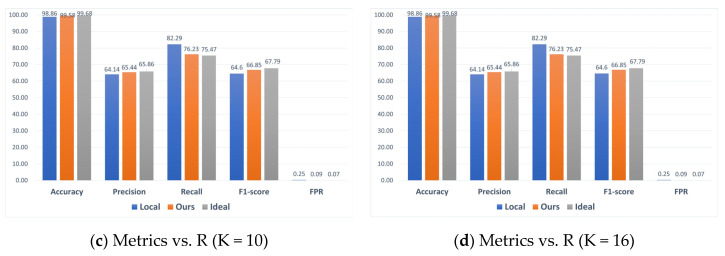
Comparison of local, ideal, and ours under four scenarios on BoT-IoT datasets.

Figure 11 shows the relevant results on the BoT-IoT dataset, where the federated learning scheme outperforms single local clients in terms of accuracy, precision, F1-score, and FPR, further demonstrating the effectiveness of federated learning. 

## 6. Conclusions

This paper proposes a federated learning scheme named the DVACNN-Fed, specifically designed for Industrial Internet of Things environments. This scheme integrates convolutional neural networks with attention mechanisms, significantly improving the accuracy of identifying complex network threats and enhancing data privacy protection by introducing variational autoencoders. The experimental results demonstrate that, compared to other existing models, the computational efficiency of the DVACNN-Fed is comparable. However, the DVACNN-Fed exhibits outstanding performance in key performance metrics such as detection accuracy, precision, and false-positive rate, highlighting its strong potential in practical applications. It also achieves favorable results in privacy evaluation metrics, indicating its good privacy protection performance. The positive outcomes obtained on the two representative datasets mentioned in this paper indicate that the DVACNN-Fed possesses good generalization and scalability.

In the future, this research plans to optimize the model structure and learning algorithms further to address the growing network security threats and increasingly complex attack types. Additionally, new mechanisms to shorten training time and enhance the scalability and adaptability of the model will be explored to better adapt to the evolving industrial IoT field. Considering the potential application of federated learning in cross-domain data collaboration, future research will promote broader cross-industry collaboration while ensuring efficiency and privacy protection.

## Data Availability

In this study, we used two datasets. The TON-IoT dataset was accessed via the following link: https://research.unsw.edu.au/projects/toniot-datasets, accessed on 3 May 2024. For the BoT-IoT datasets, you can find them on the official website https://research.unsw.edu.au/projects/bot-iot-dataset, accessed on 3 May 2024.
